# Standardization of Human Calcific Aortic Valve Disease *in vitro* Modeling Reveals Passage-Dependent Calcification

**DOI:** 10.3389/fcvm.2019.00049

**Published:** 2019-04-16

**Authors:** Shinji Goto, Maximillian A. Rogers, Mark C. Blaser, Hideyuki Higashi, Lang H. Lee, Florian Schlotter, Simon C. Body, Masanori Aikawa, Sasha A. Singh, Elena Aikawa

**Affiliations:** ^1^Center for Interdisciplinary Cardiovascular Sciences, Cardiovascular Medicine, Brigham and Women's Hospital, Harvard Medical School, Boston, MA, United States; ^2^Department of Anesthesia, Critical Care and Pain Medicine, Beth Israel Deaconess Medical Center, Harvard Medical School, Boston, MA, United States; ^3^Center for Excellence in Vascular Biology, Brigham and Women's Hospital, Harvard Medical School, Boston, MA, United States; ^4^Channing Division of Network Medicine, Brigham and Women's Hospital, Harvard Medical School, Boston, MA, United States

**Keywords:** calcific aortic valve disease, valvular interstitial cells, cardiovascular calcification, ALPL/TNAP, osteogenic differentiation, proteomics, cell culture

## Abstract

Aortic valvular interstitial cells (VICs) isolated from patients undergoing valve replacement are commonly used as *in vitro* models of calcific aortic valve disease (CAVD). Standardization of VIC calcification, however, has not been implemented, which impairs comparison of results from different studies. We hypothesized that different culture methods impact the calcification phenotype of human VICs. We sought to identify the key parameters impacting calcification in primary human VICs to standardize CAVD *in vitro* research. Here we report that in calcification media containing organic phosphate, termed osteogenic media (OM), primary human VICs exhibited a passage-dependent decrease in calcification potential, which was not observed in calcification media containing inorganic phosphate, termed pro-calcifying media (PM). We used Alizarin red staining to compare the calcification potential of VICs cultured in OM and PM between the first and fourth passages after cell isolation from human CAVD tissues. Human VICs showed consistent Alizarin red stain when cultured with PM in a passage-independent manner. VICs cultured in OM did not exhibit consistent calcification potential between donors in early passages and consistently lacked positive Alizarin red stain in late passages. We performed whole cell, cytoplasmic and nuclear fractionation proteomics to identify factors regulating VIC passage-dependent calcification in OM. Proteomics cluster analysis identified tissue non-specific alkaline phosphatase (TNAP) as a regulator of passage-dependent calcification in OM. We verified an association of TNAP activity with calcification potential in VICs cultured in OM, but not in PM in which VICs calcified independent of TNAP activity. This study demonstrates that media culture conditions and cell passage impact the calcification potential of primary human VICs and should be taken into consideration in cell culture models of CAVD. Our results help standardize CAVD modeling as part of a greater effort to identify disease driving mechanisms and therapeutics for this unmet medical need.

## Introduction

Calcific aortic valve disease (CAVD) is a major cause of aortic stenosis, which results in angina, syncope, heart failure, and death (1). Despite identification of multiple risk factors and related molecular pathway alterations, there are no approved drugs for CAVD treatment (2, 3). Impairing CAVD therapeutic discovery is a lack of thoroughly validated models that accurately recapitulate human CAVD progression (2). Additionally, standardization of the few currently used CAVD *in vitro* models remains unimplemented (4). In this study we sought to identify key factors impacting the calcification potential of cultured primary human valvular interstitial cells (VICs) to advance CAVD research efforts.

Calcification forms in aortic valve interstitium in which VICs reside (5). Primary human VICs isolated from patients undergoing valve replacement surgery or from transplanted hearts offer an important research tool to better understand mechanisms of CAVD and for exploring potential therapeutic interventions. Surgical tissue specimens are of great value as they represent the rare opportunity to analyze human CAVD, as many aortic valve replacement surgeries will be eventually substituted by transcatheter valve replacement procedures. *In vitro* induction of VIC calcification often uses media containing a combination of dexamethasone, organic phosphate (β-glycerophosphate), and ascorbic acid, referred to here as osteogenic media (OM) (6). A wide range of Alizarin red stain, from strong to almost no calcification staining, has been reported for human VICs when cultured in OM without the addition of other calcification-promoting compounds such as pro-inflammatory stimuli (7–10). Dexamethasone, a component of OM, was initially demonstrated to promote bone osteoblast differentiation (11). OM was subsequently used to induce calcification in vascular smooth muscle cell cultures by promoting osteogenic differentiation (12). OM induces expression of osteogenic markers, including runt related transcription factor 2 (*RUNX2*), osteocalcin (*BGLAP*), tissue non-specific alkaline phosphatase (*ALPL/*TNAP) and TNAP activity in VICs (13). TNAP regulates calcification by two mechanisms (14): (1) TNAP hydrolyzes and thereby reduces pyrophosphate, a calcification inhibitor; (2) in the case of OM, TNAP hydrolyzes β-glycerophosphate to inorganic phosphate that is incorporated into nascent hydroxyapatite crystals forming cardiovascular calcifications. In addition to OM, media containing high inorganic phosphate and ascorbic acid, referred here as pro-calcifying medium (PM), induces calcification. PM induces calcification, visualized by Alizarin red stain in human VICs (9, 15, 16). It is unclear whether OM or PM systems model different pathologies of human CAVD, as such further work examining VIC calcification potential and mechanisms in these media conditions is required. The relevance of these commonly used means to model CAVD *in vitro* to cases with additional CAVD-associated complications remains to be determined. With high inorganic phosphate, PM may be well-suited for modeling CAVD associated with altered phosphate metabolism, such as in chronic kidney disease patients (17, 18). A side-by-side comparison of calcification potential in human VICs over several cell culture passages in organic phosphate-containing OM and inorganic phosphate-containing PM has not yet been reported. We therefore performed this task as a first effort to help standardize CAVD research for a better understanding of the pathological processes that lead to VIC calcification and aortic stenosis.

## Methods

### Human CAVD Cell Culture

Human aortic valve leaflets were obtained from aortic valve replacement surgeries for severe aortic valve stenosis (Brigham and Women's Hospital approved IRB protocol number: 2011P001703). Written informed consent was obtained for the human tissues used in this study. VICs were isolated from the leaflets using sequential collagenase digestions in the same manner which we and others have previously described (9, 15, 16). Briefly, both sides of the leaflet were scratched by razor blade to remove the endothelial cells. After cutting into 1–2 mm cubes, tissue pieces were digested using 1 mg/mL collagenase (Sigma-Aldrich, St. Louis, MO) in DMEM (Thermo Fisher Scientific, Waltham, MA) for 1 hour at 37°C and gently mixing every 20 minutes. The cells isolated in the first digestion potentially include endothelial cells, which were washed out by DMEM and discarded. Next, tissue pieces were further digested using 1 mg/mL collagenase for 3 hours and isolated VICs were collected by centrifugation and plated in 75 cm^2^ culture flasks. Isolated VICs were cultured in DMEM supplemented with 10% FBS and 1% penicillin/streptomycin (Corning, Corning, NY) until cells were >90% confluent (~9–14 days later). Cells were then trypsinized and plated for each experiment at a density 3.0 × 10^4^ cells/0.9 cm^2^ (1 well of a 48-well plate), which resulted in 100% confluency. Remaining cells were subsequently passaged at a 1:3 dilution.

### Quantification of Aortic Valve Tissue Calcification

Calcification content in human aortic valve leaflets was evaluated as previously described (19), by quantifying the area of the calcified tissue (indicated by calcified nodules) in valve leaflets with the use of NIH ImageJ software (Bethesda, MD).

### VIC Calcification Staining

Human VICs plated in 48-well plates were cultured in control normal medium (termed NM) containing DMEM with 5% FBS, or osteogenic medium (termed OM) containing, DMEM with 5% FBS, 10 nmol/L dexamethasone (MP Biomedicals, Santa Ana, CA), 10 mmol/L β-glycerophosphate (EMD Millipore, Burlington, MA), and 50 μg/mL L-ascorbic acid (Sigma-Aldrich), or pro-calcifying medium (termed PM) containing, DMEM with 5% FBS, 2 mmol/L NaH_2_PO_4_ (Sigma-Aldrich) (pH 7.4) and 50 μg/mL L-ascorbic acid) for 28 days. To assess TNAP calcification dependency, 1 μmol/L TNAP inhibitor (EMD Millipore) or 0.01% DMSO vehicle control as added to OM and PM. Calcium deposition was stained using 2% Alizarin red (Lifeline Cell Technology, Frederick, MD). Briefly, cells were fixed by 10% formalin for 15 minutes and washed twice with distilled water. After adding Alizarin red solution, cells were stained for 15 minutes at room temperature. Excess stain was washed twice with distilled water. Alizarin red stain was quantified by extracting the stain with 100 mmol/L cetylpyridinium chloride (bioWORLD, Dublin, OH) and measuring the absorbance at 540 nm.

### TNAP Activity

Human VICs plated in 48-well plates were cultured in NM, OM, and PM for 14 days. TNAP activity was measured using a TNAP activity assay kit (BioVision, Inc., Milpitas, CA), according to the manufacturer's protocol. Briefly, cells were washed with PBS and lysed with TNAP assay buffer on ice. After centrifugation, the supernatant of the cell lysate was used for TNAP activity assessment. TNAP enzymatic reaction was performed by incubating samples at 37°C for 1 hour. TNAP activity was normalized by protein concentration measurements obtained using a BCA protein assay kit (Thermo Fisher Scientific).

### RNA Analysis

Human VICs plated in 24-well plates were cultured in NM or OM for 14 days. TRIzol reagent (Thermo Fisher Scientific) was used to isolate RNA. cDNA was generated from the RNA using Quanta qScript cDNA Synthesis Kit (Quantabio, Beverly, MA). mRNA levels were quantified by TaqMan real-time PCR with the following probes (Thermo Fisher Scientific): *GAPDH* (Hs02758991_g1), *TP53* (Hs01034249_m1), *CDKN1A* (Hs00355782_m1), and *CDKN2A* (Hs00923894_m1). mRNA levels were calculated by the ΔΔCt method normalizing to *GAPDH*.

### Proteomics

Human VICs plated in 6-well plates were cultured in NM, OM, and PM for 14 days. Cells were trypsinized and resuspended in 1 mL PBS. Cell suspensions were separated into two parts, 0.2 and 0.8 mL, for whole cell lysate and nuclear/cytoplasmic fractionation, respectively. To obtain whole cell lysate, 0.2 mL of the cell suspensions were centrifuged at 500 times gravity for 5 min and the pellets were lysed with 100 μL RIPA buffer (Thermo Fisher Scientific) containing 1% protease inhibitor cocktail (Sigma-Aldrich). Nuclear/cytoplasmic fractionation was performed using NE-PER Nuclear and Cytoplasmic Extraction Reagents (Thermo Fisher Scientific). Whole cell lysate and fractionated cell lysate protein were extracted and digested by methanol-chloroform and trypsin (Promega, Madison, WI). 5 μg protein was used per sample. The tryptic peptides were desalted using Oasis Hlb 1cc (10 mg) columns (Waters, Milford, MA), and dried with a tabletop speed vacuum (Eppendorf AG, Germany). After re-suspension in 40 μl of 5% mass spectrometry (MS) grade acetonitrile (Thermo Fisher Scientific) and 0.5% formic acid (Thermo Fischer Scientific) in water (Thermo Fisher Scientific). Tryptic peptide samples were analyzed on an Orbitrap Fusion Lumos mass spectrometer fronted with an EASY-Spray Source (heated at 45°C) and coupled to an Easy-nLC1000 HPLC pump (Thermo Fisher Scientific). Peptides were subjected to a dual column set-up: an Acclaim PepMap RSLC C18 trap analytical column, 75 μm × 20 mm (pre-column), and an EASY-Spray LC column, 75 μm × 250 mm (Thermo Fisher Scientific). The analytical gradient was run at 300 nl/minute from 5 to 21% Solvent B (acetonitrile/0.1 % formic acid) for 75 minutes, 21 to 30 % Solvent B for 15 minutes, followed by 10 minutes of 95% Solvent B. Solvent A was water/0.1% formic acid. The acetonitrile and water were MS-grade. The Orbitrap analyzer was set to 120 K resolution, and the top N precursor ions in 3 seconds cycle time within a scan range of 375–1,500 m/z (60 s dynamic exclusion enabled) were subjected to collision induced dissociation (CID; collision energy, 30%; isolation window, 1.6 m/z; AGC target, 1.0 e4). The ion trap analyzer was set to a rapid scan rate for peptide sequencing (MS/MS).MS/MS data were queried against the Human UniProt database (downloaded on August 01, 2014) using the SEQUEST-HT search algorithm, via the Proteome Discoverer Package (version 2.2, Thermo Fisher Scientific). Trypsin was set as the digestion enzyme in the software, allowing up to four missed cleavages, using 10 ppm precursor tolerance window and 0.6 Da fragment tolerance window. Oxidation of methionine was set as a variable modification, and carbamidomethylation of cysteine was set as a fixed modification. The peptide false discovery rate was calculated using the Percolator algorithm provided by Proteome Discoverer and peptides were filtered based on a 1.0% false discovery rate. Peptides assigned to a given protein group (Master protein), and not present in any other protein group, were considered as unique and used for quantification. A minimum of two unique peptides were included for each protein. In order to quantify peptide precursors detected in the MS1 but not sequenced from sample to sample, we enabled the “Feature Mapper” node. Chromatographic alignment was done with a maximum retention time shift of 10 minutes and a mass tolerance of 10 ppm. Feature linking and mapping settings were a retention time tolerance minimum of 0 minutes, mass tolerance of 10 ppm, and signal-to-noise minimum of 5. Precursor peptide abundances were based on their chromatographic intensities and total peptide amount was used for normalization.

### Proteome Clustering Analysis Using XINA Software

Analysis of proteome patterns was performed using a high-dimensional clustering software, XINA (https://bioconductor.org/packages/release/bioc/html/XINA.html) (20). The median-normalized abundance of proteins for four donors were combined into a single dataset for subsequent clustering analysis by fraction (whole cell lysate, cytoplasmic and nuclear fractions). Clusters differentiating media condition (NM, OM, and PM) between the two passages (early passage one and late passage four) were prioritized.

### Valve Tissue Proteomics Comparison

Statistically increased proteins in the VIC whole cell proteome under OM and PM conditions were compared with our previously reported normal, fibrotic, and calcified human valve tissue proteomics dataset (9). Fold changes and adjusted *P*-values of OM/NM and PM/NM ratios were obtained using Proteome Discoverer. Proteins statistically increased in OM or PM, with a fold change > 1 and *P* < 0.05, were compared to proteins enriched in three (normal, fibrotic, calcified) human valve tissue disease stages.

### Statistical Analysis

Data was analyzed using *t*-test or ANOVA with *post hoc* tests where appropriate, using PRISM software (GraphPad, San Diego, CA). *P* < 0.05 were considered significant. Error bars indicate standard deviation (SD).

## Results

### Calcification of Human VICs Was Passage-Dependent in OM but Not PM

**Figure 1:**
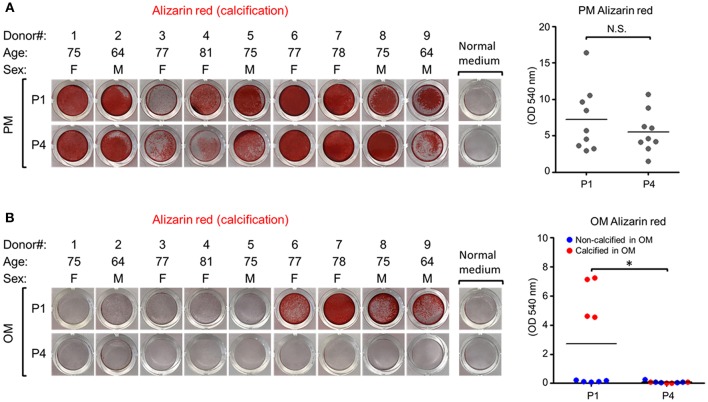
Human valvular interstitial cell (VIC) calcification was donor- and passage-dependent in osteogenic media (OM) but not passage-dependent in pro-calcifying media (PM). **(A)** Alizarin red calcification stain and quantification of human calcific aortic valve disease tissue-derived VICs in normal control media (NM) or PM at passage one (P1) and passage four (P4), or in OM **(B)**. Quantification presented as dot plots, bar indicates group mean, and for the OM dataset dots are distinguished by calcification-prone (calcified in OM, indicated by red dots) and calcification-resistant donors (non-calcified in OM, indicated by blue dots). *n* = 9 donors/media condition, F, female; M, male; N.S., non-significant; ^*^P < 0.05 analyzed by *t*-test.

**Figure 2:**
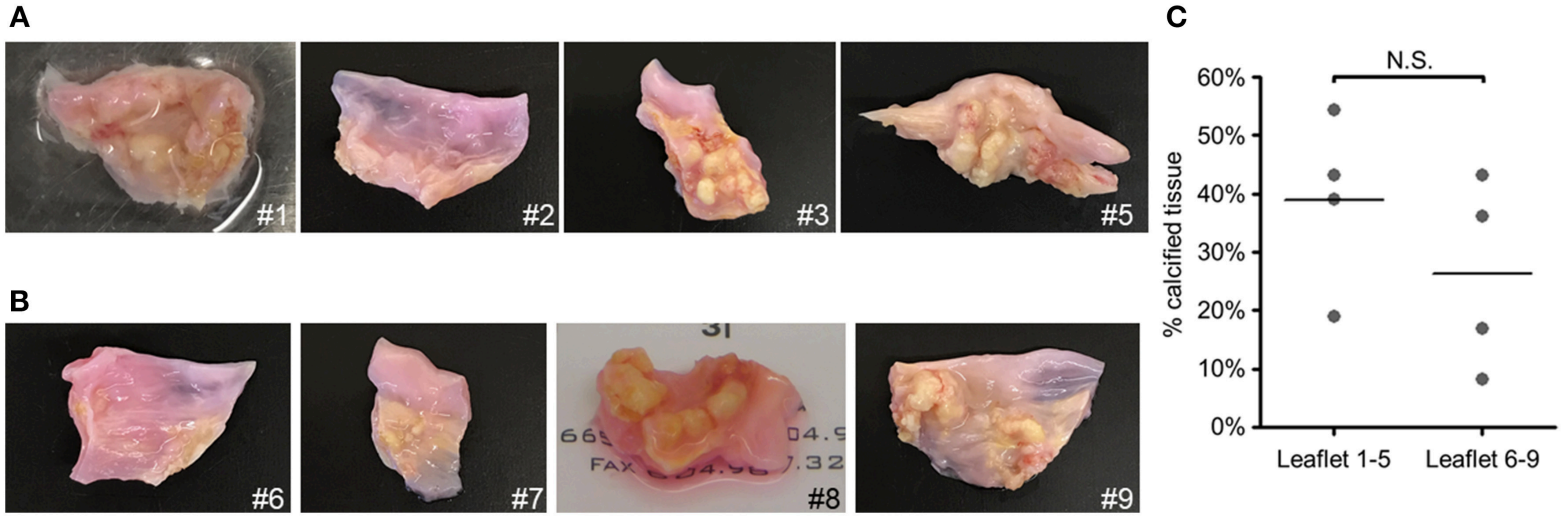
Gross calcification of valve tissues from which calcification-prone VICs were isolated was not different from that in which calcification-resistant VICs were isolated. Images of aortic valve leaflets used to obtain VICs. Tissues from which calcification-resistant VICs were obtained, leaflets 1–5 shown in **(A)**, and calcification-prone VICs were obtained, leaflets 6–9 shown in **(B)**. Calcification-prone VICs were assessed to be Alizarin red-positive in passage one when cultured in OM, whereas calcification-resistant VICs did not calcify under these conditions. **(C)** Quantification of the percent-positive areas of calcified leaflets (indicated by calcified nodules on the images). 8 of 9 donors shown (image was not collected for donor 4); *n* = 4 donors per leaflet group, N.S., non-significant.

To assess whether calcification potential of human VICs was passage dependent, we measured VIC calcification at the first passage (P1) and fourth passage (P4) post-isolation. These passages were selected based on a prior report of passage-dependent calcification in porcine VICs that have decreased calcification potential after three passages (13). In PM, VICs readily calcified in all nine donors assessed and no differences were observed in Alizarin red calcification stain between P1 and P4 (Figure 1A). In OM, four of nine VIC donors were calcification-prone and had noticeable Alizarin red stain at P1 (Figure 1B); however, calcification in these four donors was negligible by P4. No significant differences in amount of calcified nodules were observed in the tissues from which calcification-resistant VICs (Figure 2A) were isolated compared to those from which calcification-prone VICs (Figures 2B,C) were isolated. This data demonstrates that OM-cultured VICs exhibit donor-to-donor variation and passage-dependent calcification, whereas as PM-cultured VICs do not exhibit passage-dependent calcification.

### Senescence-Associated mRNA Levels in Human VICs Were Not Altered by OM

**Figure 3:**
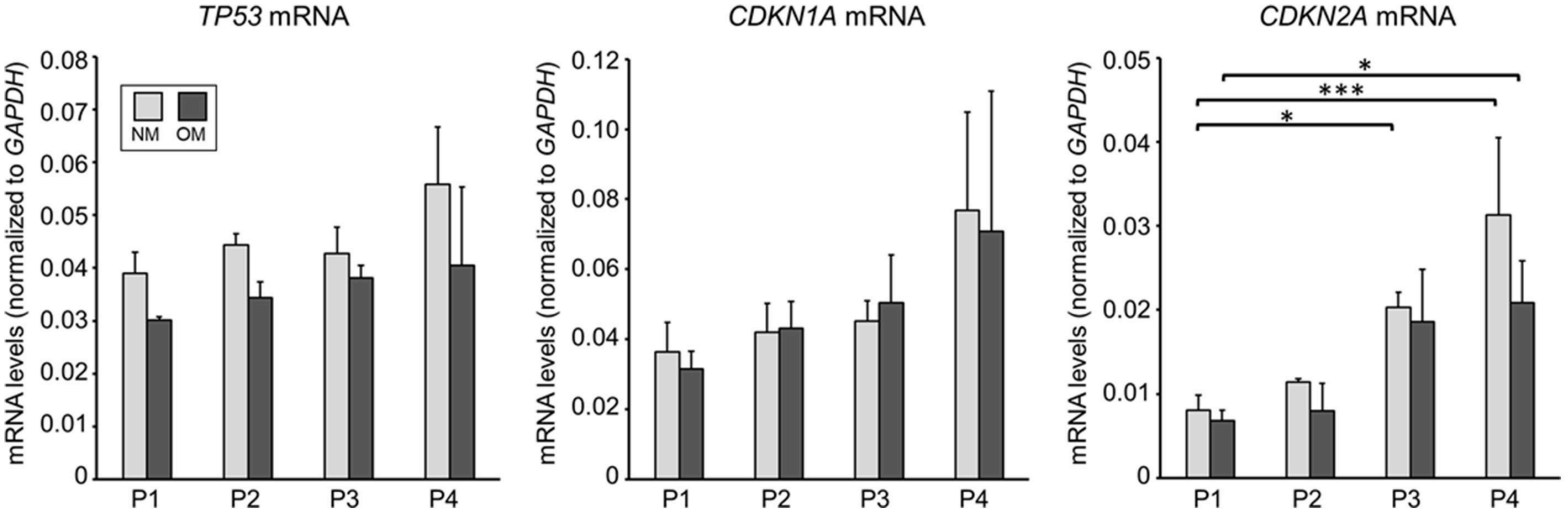
Senescence-associated mRNA levels in human VICs were not altered by OM. mRNA levels of tumor promoting protein p53 (*TP53*), cyclin dependent kinase inhibitor 1A (*CDKN1A*), and cyclin dependent kinase inhibitor 2A (*CDKN2A*) in calcific aortic valve disease tissue-derived VICs cultured in control normal media (NM) or OM. *n* = 3 donors, P1–4 = passage 1–4, ^*^P < 0.05, ^***^P < 0.001 analyzed by ANOVA with Bonferroni's multiple comparison test, error bars indicate SD.

As we observed passage-dependent calcification in OM we sought to assess whether this change was due to altered cellular senescence. Supporting a role of cellular senescence in cardiovascular calcification, prelamin A accumulation induces DNA damage signaling that acts upstream of senescence-associated phenotypes and calcification in vascular smooth muscle cells (21). Increased cell passage induces cellular senescence that in turn might impact VIC calcification potential. We measured mRNA levels of senescence-associated genes (22), including tumor promoting protein p53 (*TP53*), cyclin dependent kinase inhibitor 1A (*CDKN1A*), and cyclin dependent kinase inhibitor 2A (*CDKN2A*) in VICs cultured in OM. *CDKN2A*, but not *TP53* and *CDKN1A* mRNA levels increased between P1 and P4; however, no significant differences were observed in these senescence-associated mRNA levels in OM compared to cells cultured in NM (Figure 3). Lack of alterations in cellular senescence-associated mRNA levels in OM suggest that some other mechanisms likely regulated our observed passage-dependent calcification in VICs cultured in OM.

### TNAP Abundance Associated With Donor and Passage-Dependent Calcification in OM

**Figure 4:**
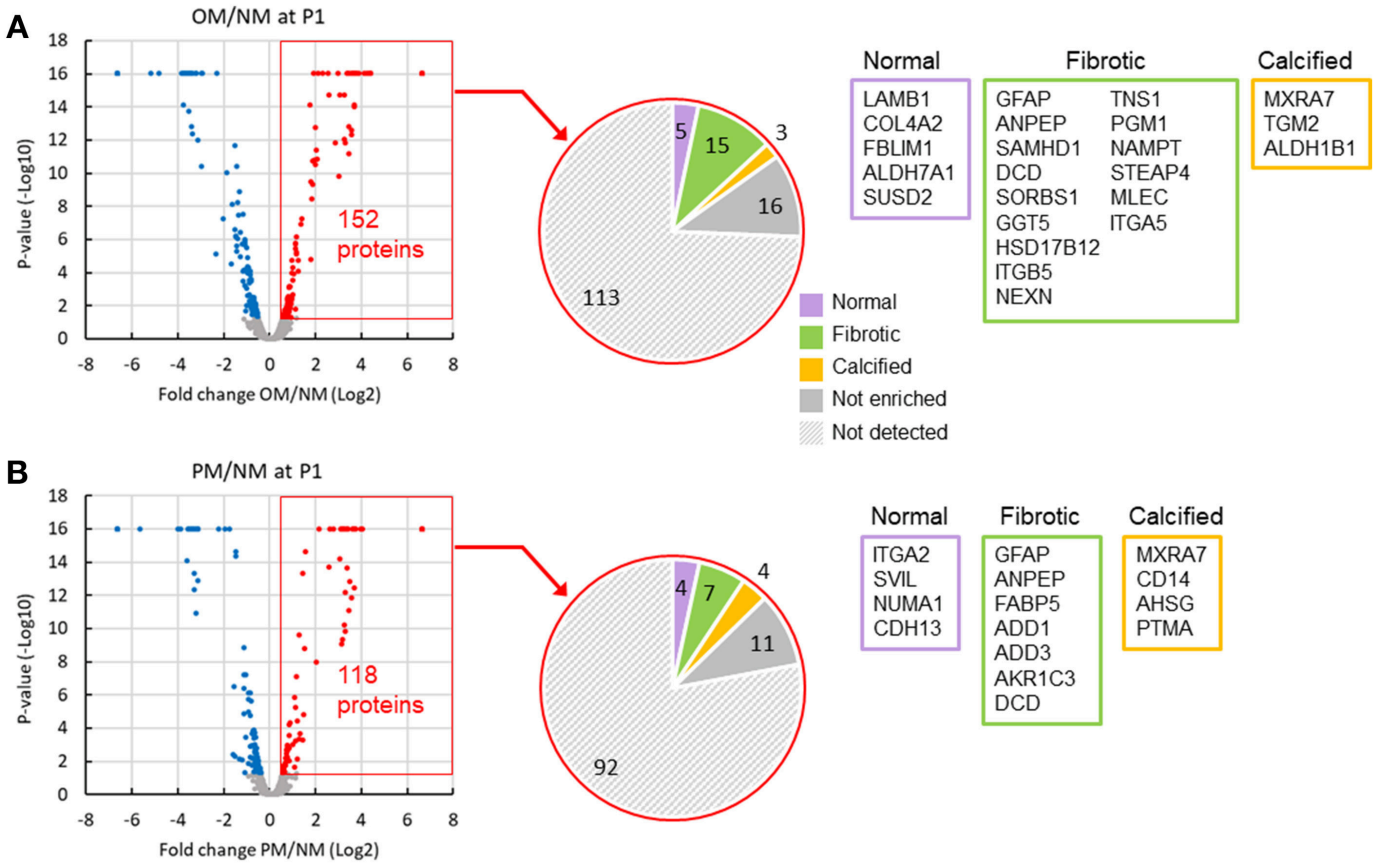
Comparison of VIC and valve tissue proteomics datasets indicated some shared regulated proteins. Volcano plots of increased (red) and decreased (blue) passage one VIC proteins in OM **(A)** and PM **(B)** after 2 weeks in culture. Comparison of increased VIC proteins (152 in OM and 118 in PM) to valve tissue disease stage proteomics dataset shown in pie charts. List of gene names for proteins increased in OM or PM, which were also found to be enriched in normal (purple box), fibrotic (green box), and calcified (orange box) human aortic valve tissues. Not enriched indicates proteins increased in VICs that were not found to be enriched in any of the three valve tissue disease stages. Not detected indicates proteins not detected in the untargeted valve tissue proteomics dataset; *n* = 4 VIC donors and 9 human valve tissue donors.

To identify passage-dependent abundance or localization changes in positive or negative calcification regulators in human VICs, we performed proteomic analysis on whole cell lysates, and nuclear and cytoplasmic fractions from VICs cultured in NM, OM, or PM (Supplemental Data File 1). To demonstrate the relevance of our *in vitro* dataset to *in vivo* conditions, we first compared the proteome of VICs cultured in OM and PM to the proteome of normal, fibrotic, and calcified valve tissue disease stages using our previously reported human tissue dataset (9). Fifteen proteins were found to be increased in OM (Figure 4A) and seven in PM (Figure 4B) that were also enriched in fibrotic human valve tissue, including glial fibrillary acidic protein (GFAP), alanyl aminopeptidase (ANPEP), and dermcidin (DCD) in both OM and PM. Three proteins were increased in OM (Figure 4A) and four in PM (Figure 4B) that were also enriched in calcified human valve tissue, including matrix remodeling associated 7 (MXRA7) in both OM and PM. This data supports that there is some overlap with both OM and PM conditions, and between human VIC cell culture models and diseased human valve tissues.

**Figure 5:**
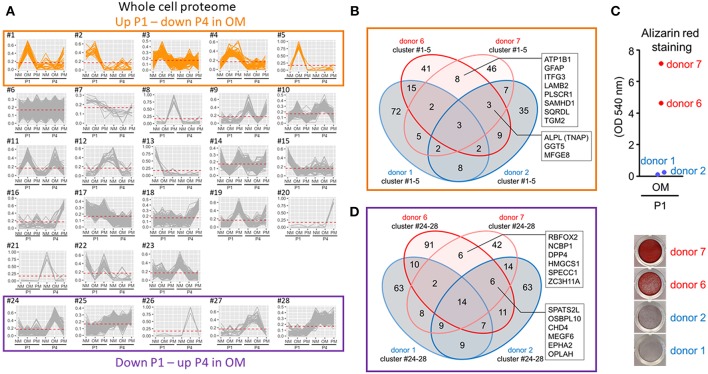
Valvular interstitial cell whole cell proteomics identified TNAP as a regulator of donor- and passage-dependent calcification in OM. **(A)** XINA clustering analysis of protein abundances in NM, OM, and PM. Clusters with increased protein abundance at passage one (P1) and decreased abundance at passage four (P4) in OM indicated in orange (clusters #1–5). Clusters with decreased protein abundance at P1 and increased abundance at P4 in OM indicated by purple box (clusters #24–28); *n* = 4 donors. **(B)** Venn diagram showing total number of proteins detected for the four donors analyzed (donors 1, 2, 6, 7) and found in the five identified clusters in which abundance was increased at P1 and decreased at P4 in OM. Calcification-prone donors (calcified at P1 in OM; donors 6 and 7) indicated by red color, and calcification-resistant donors (did not calcify at P1 in OM; donors 1 and 2) indicated by blue color. Proteins detected in the five clusters of the two calcification-prone but not in the calcification-resistant donors indicated: sodium/potassium-transporting ATPase subunit beta-1 (ATP1B1), glial fibrillary acidic protein (GFAP), family with sequence similarity 234 member A (ITFG3), laminin subunit beta 2 (LAMB2), phospholipid scramblase 1 (PLSCR1), SAM, and HD domain containing deoxynucleoside triphosphate triphosphohydrolase 1 (SAMHD1), sulfide:quinone oxidoreductase (SQRDL), and transglutaminase 2 (TGM2). Proteins detected in the five clusters in the two calcifying donors and one of the two non-calcifying donors indicated: tissue non-specific alkaline phosphatase activity (TNAP), gamma-glutamyltransferase 5 (GGT5), milk fat globule-EGF 8 (MFGE8). **(C)** Alizarin red staining at P1 for the four donors used in proteomics analysis (data also included, in part, in Figure 1B). **(D)** Venn diagram showing total number of proteins detected for the four donors analyzed (donors 1, 2, 6, 7) and found in the five identified clusters in which abundance was decreased at P1 and increased at P4 in OM.

To assess protein differences in calcification-prone and resistant donors, we used four VIC donors for proteomics analysis: two calcification-resistant donors (“donor 1” and “donor 2” in Figure 1B) that did not show positive Alizarin red stain in OM at P1, and two calcification-prone donors (“donor 6” and “donor 7” in Figure 1B) that were Alizarin red positive. Using XINA software (20), we performed high-dimensional cluster analysis of the combined four donors' VICs cultured in NM, OM, or PM at P1 and P4 to compare media-dependent and passage-dependent changes. The variance in the whole cell proteome was best explained by 28 protein abundance-based clusters (Figure 5A). To determine calcification potential-regulating proteins, we focused on the clusters showing increased abundance in OM at P1 and decreased abundance in OM at P4. Five clusters showed this pattern (Figure 5A). Assessment of proteins in these five clusters showed eight proteins in calcification-prone VICs absent from the same clusters of calcification-resistant VICs (Figure 5B): sodium/potassium-transporting ATPase subunit beta-1 (ATP1B1), GFAP, family with sequence similarity 234 member A (ITFG3), laminin subunit beta 2 (LAMB2), phospholipid scramblase 1 (PLSCR1), SAM and HD domain containing deoxynucleoside triphosphate triphosphohydrolase 1 (SAMHD1), sulfide:quinone oxidoreductase (SQRDL), and transglutaminase 2 (TGM2). TNAP appeared in these five clusters in the two calcification-prone donors and in one of the calcification-resistant donors (Figure 5B). We observed association between the TNAP protein abundance pattern (Figures 5A,B) and Alizarin red stain (Figure 5C) in this set of four donors. This data along with the known roles of TNAP in promoting calcification suggest that TNAP contributed to passage- and donor-dependent calcification potential of VICs cultured in OM.

To determine if the reduced calcification observed in P4 was due to a gain of calcification inhibitors, we assessed the clusters showing decreased abundance in OM at P1 and increased abundance in OM at P4. Five clusters showed this pattern (Figure 5A). Six proteins were found in these P4 clusters from the two calcification-prone donors that were not observed in the same clusters from the calcification-resistant donors (Figure 5D). These included RNA binding Fox-1 Homolog 2 (RBFOX2), nuclear cap binding protein subunit 1 (NCBP1), dipeptidyl peptidase 4 (DPP4), 3-hydroxy-3-methylglutaryl-CoA synthase 1 (HMGCS1), cytospin-B (SPECC1), zinc finger CCCH domain-containing protein 11A (ZC3H11A). While DDP4 inhibition has been shown to suppress VIC calcification (8), none of the proteins we identified in this P4 cluster assessment are known direct calcification inhibitors. Together our data supports that reduced TNAP activity is a key regulatory factor in the suppression of passage-dependent calcification, compared to alternative pathways associated with increased calcification inhibitors.

### Proteomics Identified Novel Proteins Associated With VIC Calcification in OM

**Figure 6:**
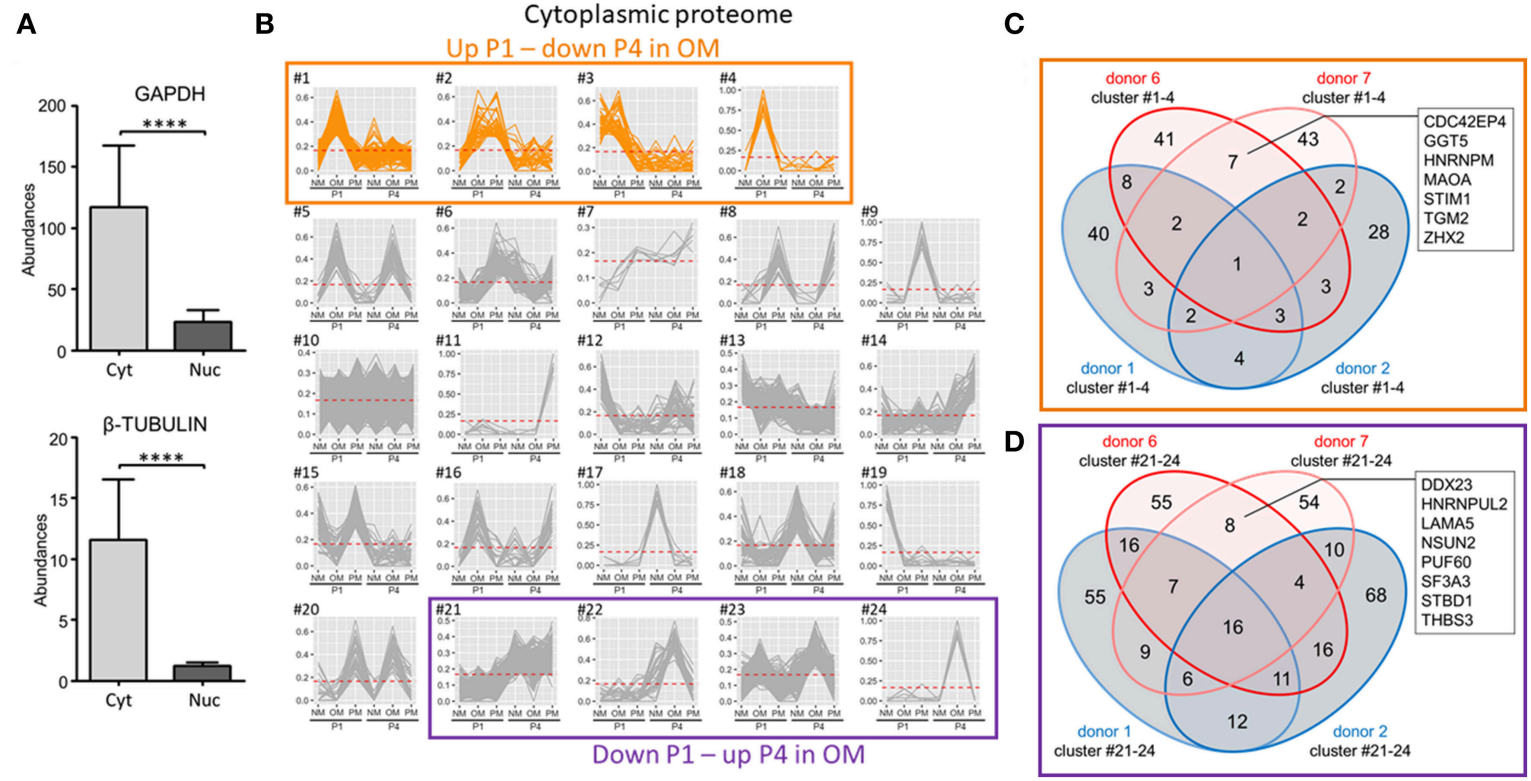
Cytoplasmic-enriched fraction proteomics identified novel proteins associated with VIC calcification in OM. **(A)** Confirmation of cytoplasmic enrichment using two cytoplasm markers, glyceraldehyde 3-phosphate (GAPDH) and tubulin-β; *n* = 4 donors/fraction, ^****^P < 0.0001 analyzed by *t*-test, error bars indicate SD. **(B)** XINA clustering analysis of protein abundances in NM, OM, and PM. Clusters with increased protein abundance at passage one (P1) and decreased abundance at passage four (P4) in OM indicated (clusters #1–4). Clusters with decreased protein abundance at P1 and increased abundance at P4 in OM indicated by purple box (clusters #21–24); *n* = 4 donors. **(C)** Venn diagram showing total number of proteins detected for the four donors analyzed (donors 1, 2, 6, 7) and found in the four identified clusters in which abundance was increased at P1 and decreased at P4 in OM. Calcification-prone donors (calcified at P1 in OM; donors 6 and 7) indicated by red color, and calcification-resistant donors (did not calcify at P1 in OM; donors 1 and 2) indicated by blue color. Proteins detected in the four clusters of the two calcification-prone but not in the calcification-resistant donors indicated: CDC42 effector protein 4 (CDC42EP4), gamma-glutamyltransferase 5 (GGT5), heterogeneous nuclear ribonucleoprotein M (HNRNPM), monoamine oxidase A (MAOA), stromal interaction molecule 1 (STIM1), transglutaminase 2 (TGM2), and zinc fingers and homeoboxes 2 (ZHX2). **(D)** Venn diagram showing total number of proteins detected for the four donors analyzed (donors 1, 2, 6, 7) and found in the four identified clusters in which abundance was decreased at P1 and increased at P4 in OM.

We next demonstrated the cytoplasmic fraction proteome to have clear cytoplasmic protein enrichment by comparing two cytoplasm markers, glyceraldehyde 3-phosphate (GAPDH) and tubulin-β, with the nuclear fraction proteome (Figure 6A). As with our analysis of the whole cell proteome, we assessed cytoplasmic fraction proteins increased at P1 and decreased at P4 in OM. In the case of the cytoplasmic fraction, 24 clusters were generated with four of these clusters having patterns that increased at P1 and decreased at P4 in OM (Figure 6B). In these four clusters, seven proteins were found in the two calcification-prone donors but not in the same clusters of the two calcification-resistant donors in OM (Figure 6C): CDC42 effector protein 4 (CDC42EP4), gamma-glutamyltransferase 5 (GGT5), heterogeneous nuclear ribonucleoprotein M (HNRNPM), monoamine oxidase A (MAOA), stromal interaction molecule 1 (STIM1), transglutaminase 2 (TGM2), and zinc fingers and homeoboxes 2 (ZHX2). To determine if the reduced calcification observed in P4 was due to a gain of calcification inhibitors, we also assessed the cytoplasmic clusters showing decreased abundance in OM at P1 and increased abundance in OM at P4. Four clusters showed this pattern (Figure 6B). Eight proteins were found in these P4 clusters in the calcification-prone but not calcification-resistant donors (Figure 6D), including DEAD-box helicase 23 (DDX23), heterogeneous nuclear ribonucleoprotein U Like 2 (HNRNPUL2), laminin subunit alpha 5 (LAMA5), NOP2/Sun RNA methyltransferase family member 2 (NSUN2), poly(U)-binding-splicing factor 60 (PUF60), splicing factor 3a subunit 3 (SF3A3), starch binding domain-containing protein 1 (STBD1), thrombospondin 3 (THBS3). None of these eight proteins are known direct inhibitors of calcification.

**Figure 7:**
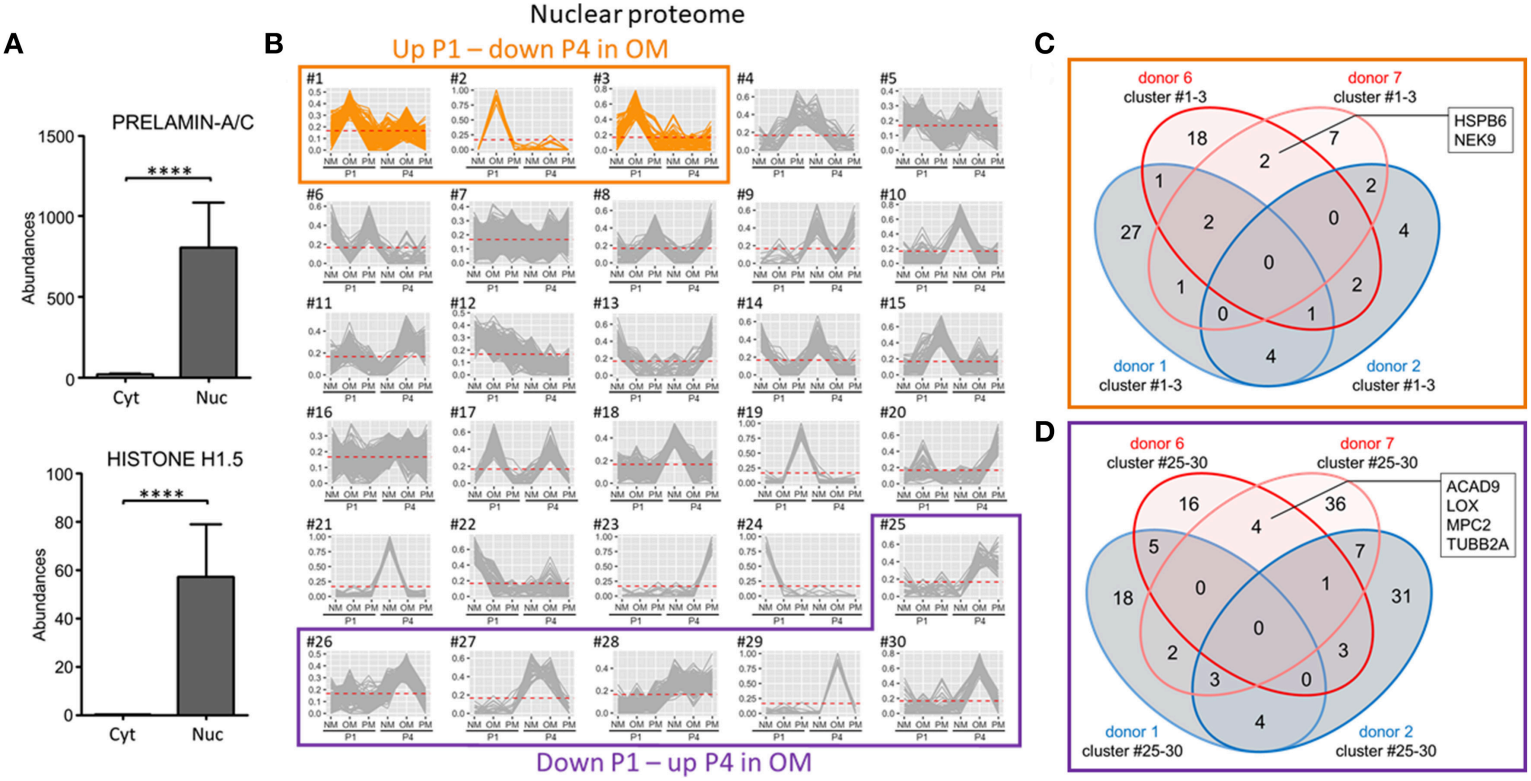
Nuclear-enriched fraction proteomics identified novel proteins associated with VIC calcification in OM. **(A)** Confirmation of nuclear enrichment using two nuclear markers, prelamin-A/C and histone H1.5; *n* = 4 donors/fraction, ^****^P < 0.0001 analyzed by *t*-test, error bars indicate SD. **(B)** XINA clustering analysis of protein abundances in NM, OM, and PM. Clusters with increased protein abundance at passage one (P1) and decreased abundance at passage four (P4) in OM indicated (clusters #1–3). Clusters with decreased protein abundance at P1 and increased abundance at P4 in OM indicated by purple box (clusters #25–30); *n* = 4 donors. **(C)** Venn diagram showing total number of proteins detected for the four donors analyzed (donors 1, 2, 6, 7) found in the three identified clusters in which abundance is increased at P1 and decreased at P4 in OM. Calcification-prone donors (calcified at P1 in OM; donors 6 and 7) indicated by red color, and calcification-resistant donors (did not calcify at P1 in OM; donors 1 and 2) indicated by blue color. Proteins detected in the three clusters of the two calcification-prone but not in the calcification-resistant donors indicated: heat shock protein family B (HSPB6), NIMA related kinase 9 (NEK9). **(D)** Venn diagram showing total number of proteins detected for the four donors analyzed (donors 1, 2, 6, 7) and found in the six identified clusters in which abundance was decreased at P1 and increased at P4 in OM.

Nuclear fraction proteome was demonstrated to have clear nuclear protein enrichment by comparing two nuclear markers, prelamin-A/C and histone H1.5 with the cytoplasmic fraction proteome (Figure 7A). Of the 30 clusters defining the variance in the nuclear proteome, three had patterns that increased at P1 and decreased at P4 in OM (Figure 7B). Two proteins were found in these three clusters in the two calcification-prone donors but not in the same clusters of the two calcification-resistant donors in OM (Figure 7C): heat shock protein family B (HSPB6), and NIMA related kinase 9 (NEK9). To determine if the reduced calcification observed in P4 was due to a gain of calcification inhibitors, we also assessed the nuclear clusters showing decreased abundance in OM at P1 and increased abundance in OM at P4. Six clusters showed this pattern (Figure 7B). Four proteins were found in these P4 clusters in the calcification-prone but not calcification-resistant donors (Figure 7D), including acyl-CoA dehydrogenase family member 9 (ACAD9), lysyl oxidase (LOX), mitochondrial pyruvate carrier 2 (MPC2), tubulin beta 2a class II a (TUBB2A). While LOX inhibition has been shown to reduce high-phosphate media-induced calcification in smooth muscle cells (23), and microtubule stabilization inhibits smooth muscle cell calcification (24), none of these four proteins are known direct inhibitors of calcification. Taken together, our proteomics analysis shows that TNAP and additional novel proteins associate with donor- and passage-dependent calcification potential of VICs in OM.

### Human VIC Calcification Was TNAP Dependent in OM but Not PM

**Figure 8:**
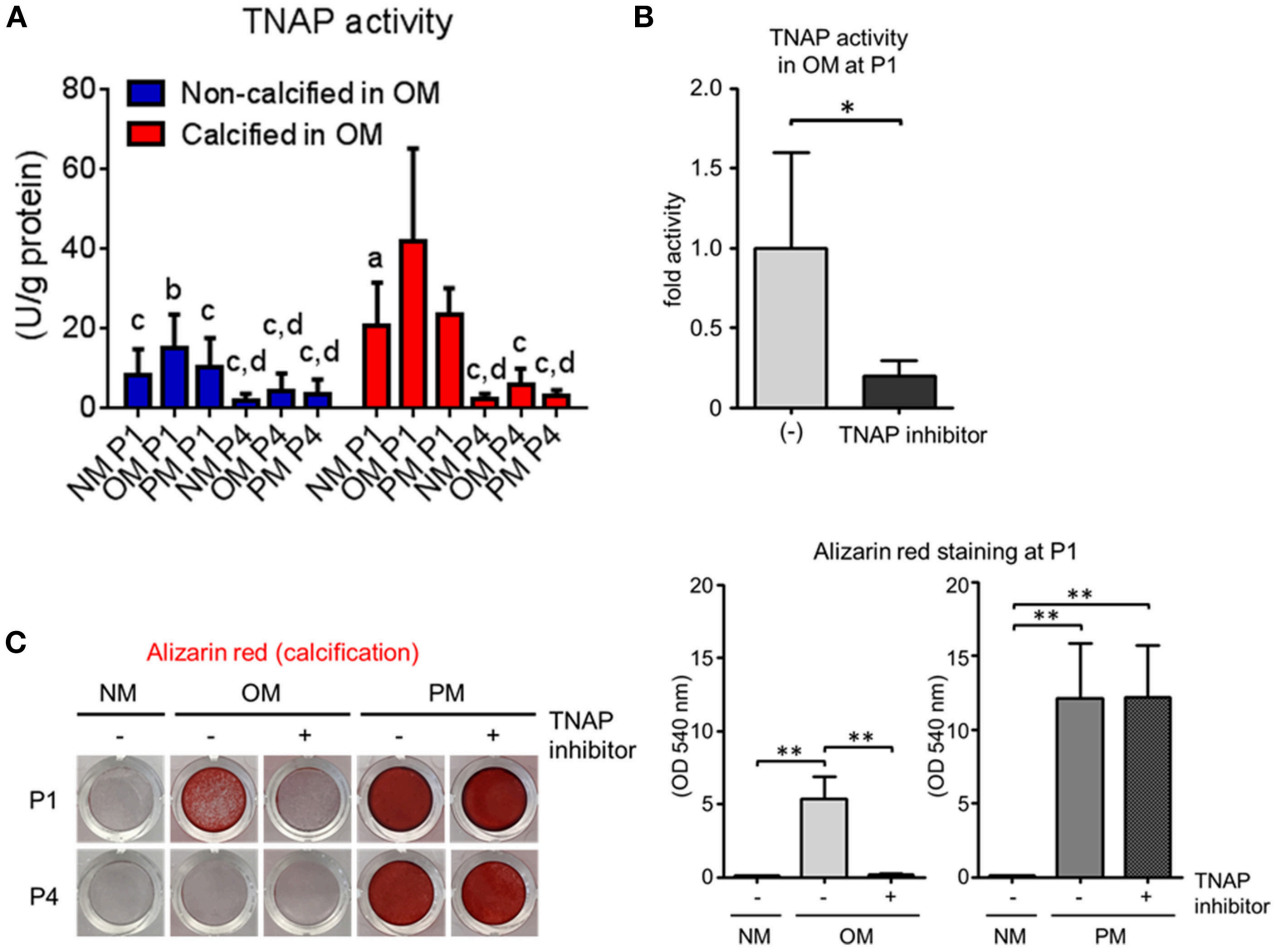
Tissue non-specific alkaline phosphatase (TNAP) regulates passage-dependent calcification in VICs in OM. **(A)** Passage one (P1) and passage four (P4) TNAP activity of calcification-prone (calcified at P1 in OM) and calcification resistant (non-calcified at P1 in OM) in NM, OM, and PM; *n* = 5 donors/group, ^a^*P* < 0.05, ^b^*P* < 0.001, ^c^*P* < 0.0001 when compared to calcified OM P1 group, and ^d^*P* < 0.05 when compared to calcified PM P1 group, analyzed by ANOVA with Tukey's multiple comparison test, error bars indicate SD. **(B)** TNAP activity at P1 in VICs in OM or OM with TNAP inhibitor; *n* = 5 donors, ^*^P < 0.05 analyzed by *t*-test, error bars indicate SD. **(C)** P1 Alizarin red stain and quantification for VICs in NM, OM, or PM with or without TNAP inhibitor; *n* = 3 donors, ^**^P < 0.01 analyzed by ANOVA with Tukey's multiple comparison test, error bars indicate SD.

As we identified TNAP abundance being increased at P1 and decreased at P4 in OM, we lastly evaluated the role of TNAP activity in VIC calcification. At P1, calcification-prone donors had significantly higher TNAP activity than calcification-resistant donors in OM (Figure 8A). Further supporting a role of TNAP in passage-dependent calcification potential, OM-induced TNAP activity decreased at P4 compared to P1 (Figure 8A). We then demonstrated that TNAP activity was required for calcification in OM by treating VICs with a TNAP inhibitor, which suppressed cellular TNAP activity (Figure 8B). Furthermore, TNAP inhibition attenuated OM-induced calcification at P1, as visualized by Alizarin red stain (Figure 8C). In contrast to OM, TNAP inhibition did not reduce calcification in PM (Figure 8C). These data demonstrate a key role of TNAP in OM- but not PM-induced VIC calcification, and further support TNAP regulation as mediating passage-dependent calcification in OM.

## Discussion

**Figure 9:**
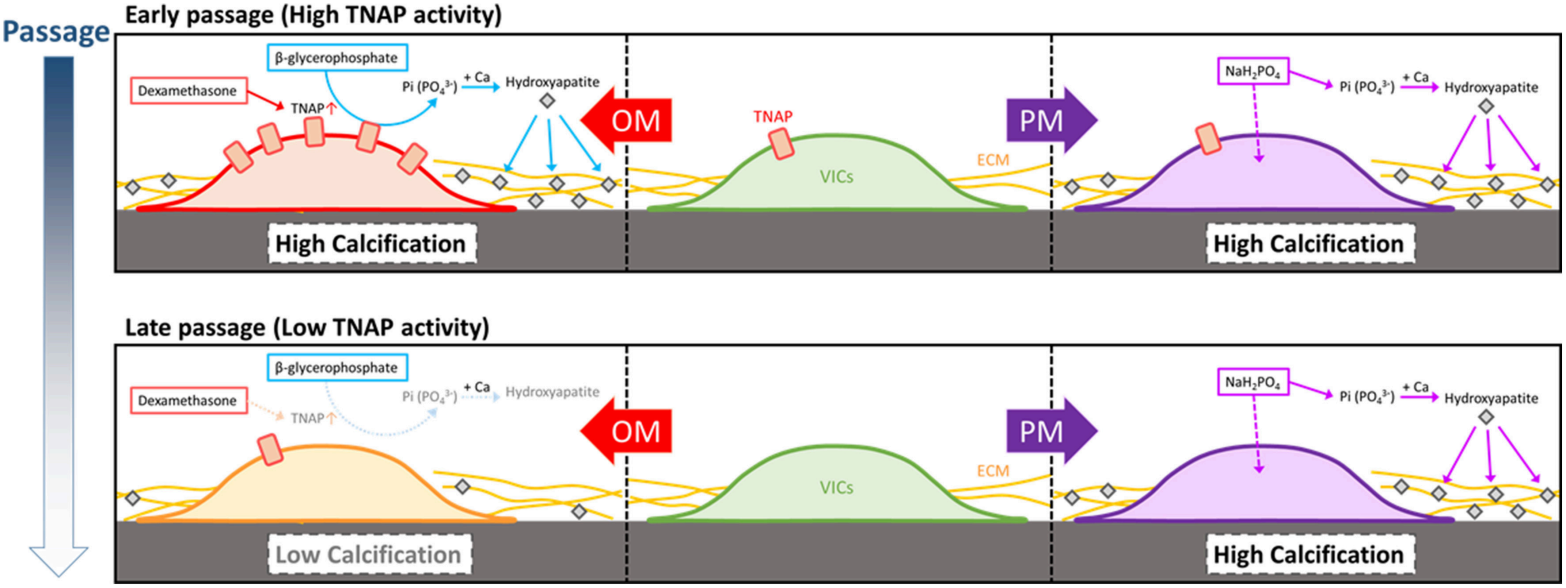
Model of OM and PM induction of VIC calcification, including passage-dependent effects. At early passage, some but not all VICs isolated from human calcific aortic valve disease donor tissues can have relatively high tissue non-specific alkaline phosphatase activity (TNAP), which is reduced by culture passaging. TNAP hydrolyzes β-glycerophosphate to inorganic phosphate that is incorporated into nascent hydroxyapatite crystals forming cardiovascular calcifications along with reducing the calcification inhibitor, pyrophosphate, making OM VIC calcification donor- and TNAP-dependent. Pro-calcifying media containing inorganic phosphate does not require TNAP to form hydroxyapatite crystals, and as such PM calcification is not suppressed by passage-dependent reductions in TNAP activity.

In a first attempt to standardize human CAVD *in vitro* research efforts we report the following novel findings: (1) calcification potential of human VICs is passage-dependent in OM but not in PM; (2) TNAP abundance and activity regulate passage-dependent calcification of VICs in OM but not in PM; (3) in addition to TNAP, several novel proteins identified in the whole cell and fractionated proteomes associate with calcification potential of VICs. We present a working model of our findings on the differences in OM- and PM-induced calcification (Figure 9). At early passage post-isolation from aortic valve tissue, some but not all VICs have relatively high TNAP allowing for calcification in OM, which is reduced by culture passaging. TNAP hydrolyzes β-glycerophosphate to inorganic phosphate, which is incorporated into nascent hydroxyapatite crystals forming calcification foci along with potentially reducing calcification inhibition via reducing non-hydrolyzed β-glycerophosphate. OM VIC calcification is donor- and TNAP-dependent due to the media phosphate source being β-glycerophosphate. PM containing inorganic phosphate does not require TNAP to form hydroxyapatite crystals, as such PM calcification is not suppressed by passage-dependent reductions in TNAP activity.

Our present study assessed VIC calcification in two-dimensional culture conditions, which is currently the most commonly used *in vitro* CAVD model. Three-dimensional cell culture has been used in a few reports (25–27). Whether our findings in two-dimensional cultures extend to three-dimensional cultures requires further study. Supporting at least some cross-over we have reported aortic valve layer-specific calcification effects in both two-dimensional (9) and three-dimensional culture conditions (27). Our previous report that human VICs calcify more strongly on the aortic side than the ventricular side (9) suggest that human CAVD VICs have different characteristics based on location within the valve leaflet. In the present study it is unclear whether calcification potential was decreased in the whole VIC population or if a ratio of calcification-prone/calcification-resistant VICs was decreased. A subpopulation of osteoprogenitors in porcine VICs significantly decreases within three passages (13). Our findings of relatively increased TNAP abundance and activity in calcification-prone VICs at early passage in OM, along with verifying OM VIC calcification to be TNAP-dependent, strongly support TNAP as a mediator of passage-dependent calcification. In addition to its role in phosphate metabolism, TNAP is also a marker of mesenchymal stem cells (28); specifically, in a subpopulation of mesenchymal stem cells with a high degree of calcification potential (29). Taking these reports together with our human VIC data it is possible that a subpopulation of calcification-prone VICs decreased during cell culture passaging, and that this population may include osteoprogenitor cells.

While both OM and PM could induce VIC calcification, PM did so in a TNAP-independent fashion. In PM, high phosphate is a likely driver of VIC calcification. Similarly, elevated phosphate uptake via the sodium-dependent phosphate cotransporter, Pit-1 mediates vascular smooth muscle cell calcification (30). High phosphate-mediated calcification is likely appropriate for modeling cases in which phosphate metabolism is altered, such as in chronic kidney disease. PM has also been used as an *in vitro* model for lipoprotein(a)-related valvular calcification (15). Lipoprotein(a) is a risk factor for CAVD (31), potentially suggesting that mechanistic findings in PM-based models could extend to a broader CAVD population. CAVD VIC calcification was TNAP-dependent in OM. TNAP activity is important for vascular smooth muscle calcification (32), particularly in OM (16, 33). The importance of TNAP activity in valvular calcification is less clear, and further work is needed to identify subpopulations of CAVD patients with TNAP-mediated calcification. A 2019 study identified TNAP in a genome-wide meta-analysis looking for CAVD susceptibility genes (34), supporting a role of TNAP in CAVD pathogenesis. Beyond TNAP, additional proteins regulate osteogenic differentiation and may play a role in our observation of passage-dependent calcification. In the whole cell proteome, ATP1B1, GFAP, ITFG3, LAMB2, PLSCR1, SAMHD1, SQRDL, and TGM2 proteins clustered differently in OM calcification-prone VICs than calcification-resistant VICs. While the mechanistic role of these novel OM calcification-associated proteins exceeds the scope of the present study, these changes indicate a difference in the proteome of calcification-prone and calcification resistant VICs. Whether this difference is due to different subpopulations of cells requires further study along with identification of any mechanistic roles these proteins may play in osteogenic regulation in VICs.

Beyond cells residing in the aortic valve, our proteomics analysis may be relevant to other calcifying cell types, as the media conditions we tested are not exclusive to VICs. This includes smooth muscle cells and bone cells, which can model calcification potential using similar media conditions to those used in our present study (11, 12, 16, 30, 33). In the case of human coronary artery and aortic smooth muscle cells cultured in OM, we have observed calcification in passages beyond P1 with higher frequency than we do using the same experimental conditions with VICs (16, 33). Similarly, we have previously demonstrated that primary human femur bone osteoblasts calcify in OM at passages later than P1 (16). Whether these *in vitro* findings indicate potential differences in valvular calcification compared to arterial calcification and bone mineralization occurring *in vivo* is unclear and requires further investigation. Our comparative proteomics analysis of VICs cultured in OM and PM showed that some proteins overlapped with proteins enriched in fibrotic and calcified valve tissue, demonstrating that there is some correlation with the cell culture models and diseased human tissue. As both OM and PM also shared some similar altered VIC proteins, in addition to OM and PM differences related to VIC passage and TNAP-dependent calcification, our data indicate that these media may also share some common signaling networks. Further in-depth computational mapping and pathway analysis of the commonalities and differences in our OM, PM, and human tissue proteome datasets will likely better reveal the CAVD pathologies most reflected by these two commonly used cell culture systems.

In conclusion, our initial attempt to standardize CAVD research efforts *in vitro* demonstrate that media culture conditions and cell passage impact the calcification potential of primary human aortic VICs and should be taken into consideration in cell culture models of CAVD. Our study serves as a starting point to further identify factors regulating calcification and to standardize the CAVD field as part of a greater effort to identify disease driving mechanisms and develop new therapeutics for this unmet medical need.

## Author Contributions

SG, MR, MB, HH, LL, FS, SB, MA, SS, and EA contributed to the collection and analysis of data. SG, MR, and EA wrote the manuscript. EA and MA contributed to the overall project supervision and funding. All authors contributed to revising and editing of the manuscript.

### Conflict of Interest Statement

The authors declare that the research was conducted in the absence of any commercial or financial relationships that could be construed as a potential conflict of interest.
